# Evaluation of the Mechanical, Thermal and Rheological Properties of Recycled Polyolefins Rice-hull Composites

**DOI:** 10.3390/ma13030667

**Published:** 2020-02-03

**Authors:** Berlinda O. Orji, Armando G. McDonald

**Affiliations:** Renewable Materials Program, Department of Forest, Rangeland and Fire Science, University of Idaho, Moscow, ID 83844-1132, USA; orji8943@vandals.uidaho.edu

**Keywords:** polyolefins, rheology, rice hulls, recycled high-density polyethylene, recycled polypropylene

## Abstract

Understanding the properties and flow characteristics of recycled polyolefins in rice hull composite blends is of importance to facilitate process optimization whilst promoting sustainability. The influence of milled rice hull particle size (<0.5 mm and <1 mm) on properties of recycled polyolefins composites was studied with major focus on recycled high-density polyethylene (rHDPE) and polypropylene (rPP) together with added maleated polymer coupling agents. Composites were compounded/extruded using a twin-screw extruder and the thermal, mechanical, and physical properties were analyzed as well as their melt flow, dynamic. and capillary rheology tests. The incorporation of the <0.5 mm rice-hulls particles enhanced the composite properties of viscosity, flexural strength, moduli, water absorption, and thermal stability for both polyolefins with rHDPE composites showing more reliable properties as compared to rPP.

## 1. Introduction

The growing awareness of the use of recycled solid waste has led to the invention of more innovative research methods in composite production with the intent of saving cost, making wood-like products with enhanced properties to promote a more sustainable environment [1,2]. A major fraction of this solid waste are polyolefins, such as high-density polyethylene (HDPE) and polypropylene (PP), which are used in industrial applications for a wide range of end consumer products [3,4]. The problem of sorting and the presence of contaminants from labels, caps, plastic bags, etc., still poses an issue during polyolefin recycling, causing a non-homogenous blend of components. Although the reuse of these polyolefins has been widely acknowledged, their continuous reprocessing under high shear force and temperature leads to a thermal degradation in structural and mechanical properties, and hence require the use of fillers to improve their performance [5,6,7]. 

Rice hulls are a lignocellulosic, renewable low-cost plant residue (20% by weight) in rice producing countries. In California alone, 4 × 10^5^ tons of hulls are produced annually and half is used to generate heat and electricity [8], leaving the remainder to be used for other applications. Rice hulls are commonly used as fuel and to a lesser extent in natural fiber composites due to its unique composition, weathering resistance, availability, biodegradability, no strength, and stability when compared to wood composites [9,10,11]. The application of rice hull composites is increasingly seen in buildings for paneling, frames, and automotive parts, but the issues of interfacial adhesion between hydrophilic fiber and hydrophobic polyolefin matrices to create homogenous composites still poses a concern. Numerous researchers have implemented the reinforcement of polyolefins with natural fibers whilst combatting the issues of interfacial compatibility. To improve a better matrix, the use of maleated graft polymer binders, silane treatment, and acetylation amongst other processes are considered [12]. The most accepted use of maleated polymer binders, maleic anhydride polypropylene (MAPP) and maleic anhydride polyethylene (MAPE), increases the polarity and adherence at the interface with reduced environmental pollution and cost [2,13,14]. 

Natural fiber composites are mostly fabricated from extrusion and injection molding, and undergo stress under deformation by applied force, hence the knowledgeable understanding of flow characteristics of these composites help determine the best ratio of fiber to recycled polyolefin for high product yield. To determine this, the process of rheology is applied. Rheology, which investigates the viscoelastic flow of composites under applied force, has been shown to help interpret interfacial adhesion performance and the degree of fiber homogeneity in composites for better product optimization [15,16]. Dynamic rheology has been employed preferably by researchers due to the low frequency applied, which reduces composite breakage compared to capillary rheology [17,18]. Several studies cover the rheology of recycled polyolefins with results showing a reduction in viscosity with multiple cycles of extrusion, tensile strength, and modulus [19,20,21,22]. 

This presented study focuses on valorizing rice hull residues and waste/recycled plastics for use in composite materials. Therefore, investigating the influence of rice hull fiber particle size on process rheology, at low and high shear rates, whilst optimizing the mechanical and physical properties in order to substitute for wood plastic composites (WPC). Rheology results were compared to melt flow rates and mechanical properties to understand the influence of composite size variation. 

## 2. Materials and Methods 

### 2.1. Materials

Commercial rice hulls (BREWCRAFT Briess, purchased at TriState Outfitters, Moscow, ID, USA) were ground using a Thomas–Wiley mill to pass through either a 0.5 or 1 mm screens. Recycled/waste rHDPE milk jugs and recycled polypropylene (rPP) (e.g., bottle caps, yoghurt and plastic containers) were obtained from the Moscow Recycling Center (Moscow, ID, USA), rinsed with water, dried (80 °C for 24 h) and then milled (<6 mm) using a plastic granulator (Sterling BP608, New Berlin, WI, USA. Commercially sourced maleated polyethylene (MAPE) (Polybond 3029, SI Group, Schenectady, NY, USA) and maleated polypropylene (MAPP) (AC950, Honeywell International Inc., Morris Plains, NJ, USA) were used as coupling agents for the composites.

### 2.2. Composite Fabrication

Milled rice hulls were oven dried at 104 °C for 24 h prior to use. Batch sizes of 500 g were prepared with rice hulls (50 wt%), and MAPE or MAPP (2 wt%) and recycled high-density polyethylene (rHDPE) or rPP (48 wt%) were extruded into ribbons (4 mm (*h*) × 50 mm (*w*)) using a co-rotating twin screw extruder (Leistritz ZSE18, Somerville, NJ, USA, LD ratio 40, 200 rpm) with a zone temperatures of 160 °C for rHDPE and 200 °C for rPP at 0.5 kg/h. The extruded ribbons were cut into 150 mm lengths and then flattened by slowly hot-pressing (PHI 30 ton press, South El Monte, CA, USA, 300 by 300 mm^2^) at 140 °C for rHDPE and 170 °C for rPP materials to a thickness of 3.2 mm. 

### 2.3. Composite Characterization

#### 2.3.1. Particle Size Measurement

Length and width of 200 rice hull particles for each screen size (<0.5 mm and <1 mm) were determined via optical microscopy (Olympus BX51 microscope fitted with a DP70 digital camera, San Diego, CA, USA) with 40× magnification, and images were analyzed using the Olympus Micro Suite software (version 3.2) [23].

#### 2.3.2. Differential Scanning Calorimetry (DSC)

DSC analysis of the composite and polyolefin samples (4–6 mg) were performed in duplicate on a Q200 DSC (TA instruments, New Caste, DE, USA) with refrigerated cooling and purged with nitrogen (20 mL/min). Scanning was performed within a range of -50 °C to 220 °C. Samples were first equilibrated at 40 °C (3 min) and then ramped to 180 °C (rHDPE) or 220 °C (rPP) (cycle 1) at 10 °C/min (held for 3 min), cooled to −50 °C (cycle 2) at −10 °C /min and reheated to corresponding temperatures (cycle 3). Data was analyzed using the TA analysis software and percent crystallinity was calculated using the equation below:

$$X_{c}=\frac{\Delta H_{m}}{\Delta H_{0}\;\times \;W_{f}}\;\times \;100\%$$

$\Delta H_{m}$ is the calculated melting enthalpy from the area under peak, $W_{f}$ is the weight fraction of polyolefin in composites and $\Delta H_{0}$ is the theoretical enthalpy of fusion for polypropylene (207.1 J/g) and polyethylene (293 J/g). 

#### 2.3.3. Melt Flow Rate 

Melt flow rate (MFR) of composite samples (4 g) was measured in triplicate using a CEAST Modular Melt Flow Indexer (Model 7024.000, Charlotte, NC, USA) at 190 °C according to ASTM D1238-01e1 through a standard die (8 mm × 2.0955 mm Ø). A load of 2.16 kg was used for recycled plastics and 15 kg for composites.

#### 2.3.4. Thermogravimetric Analysis (TGA)

The thermal degradation and stability of the composite samples (4–5 mg) was performed on a Perkin–Elmer TGA-7 instrument (Shelton, CT, USA) from 30 °C to 900 °C at 20 °C/min under nitrogen (30 mL/min). Data was analyzed using the Pyris v13.3 software.

#### 2.3.5. Thermomechanical Analysis (TMA)

Softening temperature (T_s_) and melting temperature (T_m_) of polyolefin and composite samples (2 mm (*l*) × 1.5 mm (*w*) × 1 mm (*h*)) were determined using a PerkinElmer TMA-7 instrument (Shelton, CT, USA) under nitrogen (20 mL/min) with refrigerated cooling, an applied force of 10 mN with a penetration probe mode from −30 to 180 °C at a rate of 5 °C/min. Data was analyzed using the Pyris v13.3 software.

#### 2.3.6. Tensile Testing 

Tensile tests were performed using an Instron 5500R-1132 universal testing machine (Norwood, MA, USA, 5 kN load cell and cross head speed of 5 mm/min) coupled to an extensometer (model 3542, Epsilon Technology Corp, Jackson, WY, USA) on composite and polyolefin-machined dog bone samples (nine replicates) according to the ASTM **D638** standard. Data was collected and analyzed using Bluehill v3 Instron software.

#### 2.3.7. Rheology

##### Dynamic Rotational Rheology

Dynamic rheological measurements (tanδ, viscous modulus (G″), elastic modulus (G′) and complex viscosity (η*)) on polyolefin and composite samples (2.5 mm (*h*) × 25 mm Ø) were acquired on a Bohlin CVO 100 N rheometer (East Brunswick, NJ, USA) with 25 mm Ø serrated parallel plates. Rheological measurements were determined with a plate gap of 2000 µm, 0.5% strain, frequency range of 0.01 Hz to 100 Hz, and at 190 °C. 

##### Capillary Rheology

Shear viscosity was determined using a capillary rheometer (Instron Model 3213, Norwood, MA, USA) at 190 °C connected to a Instron 5500R-1137 universal testing machine (5 kN load cell) and operating at cross head speeds of 0.6, 2, 6, 20, 60, and 100 mm/min. The barrel diameter was 9.5504 mm and two capillary dies were used (lengths of 14 mm and 27 mm with diameter of 1.4 mm Ø) to determine the effect of shear rate on the viscosity of polyolefin and composite samples. Bagley correction was also used since the L/D ratio was <200, in order to correct the influence of pressure drop on measurements. Loaded samples (7 g) were thermally equilibrated for approximately 10 min with a heating temperature variance of ±0.2 °C. Each sample was run in triplicate and data was analyzed using Bluehill v3 Instron software in conformation to the ASTM D3835-02 standard. Extrudate swell diameters were measured using a digital caliper (Mitutoyo). 

#### 2.3.8. Fourier-Transform Infrared Spectroscopy (FTIR) 

Spectra (duplicate) of polyolefin and composite samples were obtained on a Nicolet-iS5 spectrometer (Thermo-Scientific, Madison, WI, USA) with an attenuated total reflectance accessory (iD5, ZnSe). Spectra were averaged and baseline corrected using the Omnic v9.0 software. 

#### 2.3.9. Water Soak

Weight gain of circular polyolefin and composites samples (2.5 mm (*h*) by 25 mm Ø, 5 replicates) were determined after continuous soaking in water for a maximum of 100 days at 22 °C. Diffusivity was calculated using equation below:

$$D_{f}=\;\pi {\left(h/4M_{f}\right)}^{2}{\left(M/\sqrt{t}\right)}^{2}$$

where $M_{f}$ is the maximum moisture content at the end of the test, *h* is the sample thickness in meters, $M/\sqrt{t}$ is the initial slope from the plot *MC* vs $\sqrt{t}$. 

### 2.4. Data Analysis

Statistical analysis (*t*-test, paired two sample for means) was performed using Excel (Microsoft Office 2016).

## 3. Results

### 3.1. Properties of Rice-Hull Particles 

**Figure 1:**
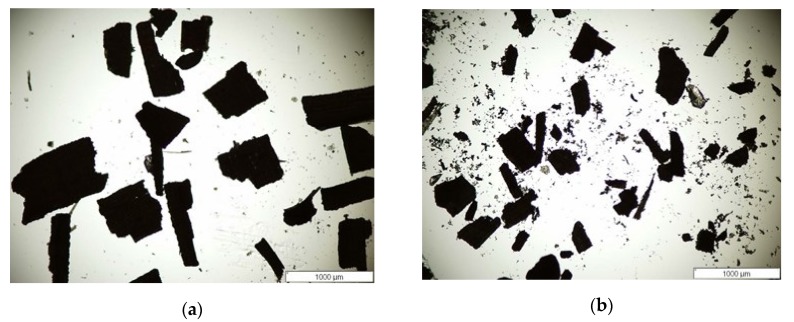
Optical micrographs of screened (**a**) <1 mm and (**b**) <0.5 mm rice hull particles (40×, scale bar 1000 µm).

The rice-hull particles were analyzed for size by optical microscopy (Figure 1). The micrographs show a range of large and fine particles. The respective average lengths of the <1 mm and <0.5 mm screened particles were 266 ± 332 µm and 174 ± 155 µm. The widths of the <1 mm and <0.5 mm screened particles were 152 ± 170 µm and 109 ± 89 µm, respectively. The calculated aspect ratios for the <1 mm and <0.5 mm screened particles were 1.8 and 1.6, respectively. The morphology shows more mixed round-rectangular shaped-fibers compared to wood fibers, which point to a less complex structure [24]. These aspect ratios are slightly higher than the reported average values of 1.2 by Raghu et al. [25], and promote better properties of the fiber as a filler.

### 3.2. Density and Tensile Properties

Tensile properties and density of polyolefins and composites were determined, and the results are given in Table 1. The density for <1 mm and <0.5 mm rHDPE composites were 1157 kg/m^3^ and 1110 kg/m^3^, respectively, as compared to 1123 kg/m^3^ and 1109 kg/m^3^ for the respective rPP composites. The presence of rice-hulls reinforcing fibers increased the density of the composite as compared to the raw polyolefins. The lower density of the <0.5 mm composites were perhaps a result of differences in packing during extrusion causing some interstitial spaces between fiber and polyolefins [26]. With the addition of rice hull fibers, statistically significant differences were observed with an increase in strength, density, and moduli, and a decrease in energy at break (EAB) for polyolefin composites at a significant level of 0.05. There were no statistical differences in moduli between the composites made with <0.5 mm and <1 mm fiber sizes for each polyolefin type. 

Tensile strength and modulus properties of rHDPE were significantly higher than the values recorded by Wang et al. [27] for HDPE (21.1 ± 0.4 MPa, 0.6 ± 0.0 GPa). The 23.8% and 83% increase in tensile strength and modulus of the rHDPE were perhaps due to different plastic grades and processing conditions, resulting in different properties. Tensile strength of the <1 mm and < 0.5 mm rHDPE composites were respectively 25.1 ± 2.9 and 26.3 ± 2.0 MPa. The tensile moduli for the composites were 2.7 ± 0.1 GPa (<1 mm) and 3.1 ± 0.5 (<0.5 mm) GPa. Chen et al. [11] obtained a lower strength value of 10.9 MPa and a modulus value of 1.446 GPa for 50 wt% rice-hull fiber with a mix of rHDPE and recycled polyethylene terephthalate (rPET) matrix. The tensile strength for the rPP composites were, 14.5 ± 0.3 MPa (<1 mm) and 16.7 ± 0.9 (<0.5 mm) MPa. The tensile moduli for the rPP composites were 2.9 ± 0.5 GPA (<1 mm) and 3.5 ± 0.2 GPa (<0.5 mm). These tensile strength for the rPP/rice hulls composites were lower than the rHDPE/rice hull composites, possibly due to thermal degradation after extrusion [28]. The EAB values for the rHDPE composites (0.64 J and 0.65 J) decreased significantly by 82% when compared to the rHDPE (3.64 J), showing a lower toughness (Table 1). The lower EAB for rPP and its composites could be attributed to an increase in brittleness as compared to rHDPE and composites. The <0.5 mm rPP composite exhibited a higher EAB showing a stronger matrix as compared to the <1 mm composite due to improved interfacial bonding.

### 3.3. Thermal Analysis

**Figure 2:**
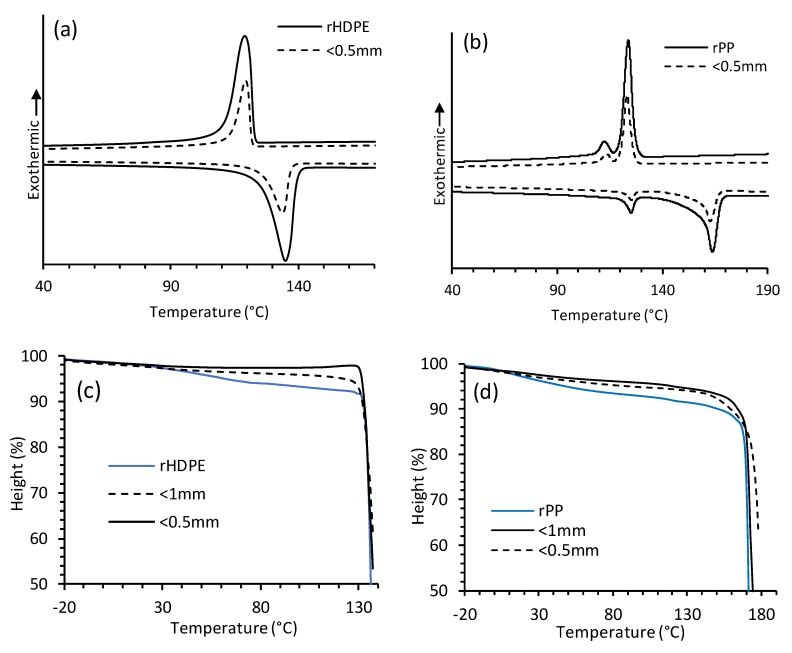
Differential scanning calorimetry (DSC) thermograms of recycled polyolefin composites for (**a**) recycled high-density polyethylene (rHDPE), (**b**) recycled polypropylene (rPP), and thermomechanical analysis (TMA) thermograms for (**c**) rHDPE and composites (**d**) rPP and composites.

To determine the melting (T_m_) and crystallization (T_c_) temperatures, DSC was performed on polyolefin and composite samples (Figure 2). Values for the melting and crystallization shift are given in Table 2. The thermograms for rHDPE and rPP are shown in Figure 2a,b. The observed T_m_ for rHDPE was 135.1 °C and a slightly lower value of 133 °C for <0.5 mm composites. Wang et al. observed a similar melt temperature for pure HDPE at 131 °C [27]. The T_c_ for rHDPE and <0.5mm composites were respectively 118.9 °C and 119.6 °C. The presence of small fibers could be attributed to a slightly faster crystallization [28]. Enthalpy of crystallization also decreased with the addition of the rice-hull fibers which restricted the movement of the polymer matrix. This contrasts the findings of reduced crystallinity with increased wood fiber content by Cui et al. [29], and agrees with the notion of a shift in crystallinity with increased temperature by Ndiayi et al. [30]. In the case of rPP, two melting and crystallinity peaks were seen, signifying either a structure change or the presence of a copolymer or impurities. The earlier (minor) T_m_ and T_c_ peaks for rPP were 125.1 °C and 112.3 °C, respectively. The main (major) T_m_ and T_c_ peaks for rPP were respectively at 163.8 °C and 123.75 °C. Also, the addition of <0.5 mm rice-hulls reduced the T_m_ and T_c_ of the composite and showed a slight shift compared to rPP. 

DSC scan peaks (T_m_) fell within the range of the TMA softening temperature (T_s_) values as shown in Table 2 for both polyolefins and composites. The more responsive TMA analysis (Figure 2c,d) shows a decrease in height and yielded T_s_ values of 130.1 °C and 156.4 °C, respectively, for rHDPE and rPP, while the <0.5 mm composites remained more stable at <1mm before softening at 131.3 °C and 161.2 °C for their composites. Glass transition temperature (T_g_) for rPP was seen at −2.2 °C by TMA. T_s_ increased slightly for the composites compared to the raw polyolefins as a result of an increase in reinforcement from the rice hull fibers. 

**Figure 3:**
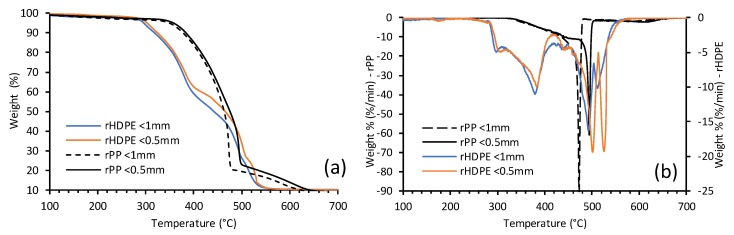
(**a**) Thermogravimetric analysis (TGA) and (**b**) differential thermogravimetric (DTG) curves for rice hull polyolefin composites.

The thermal degradation of composites was determined by TGA as shown in Figure 3a. The differential thermogravimetric (DTG) thermograms are shown in Figure 3b. The thermal degradation transition temperatures are given in Table 3. Significant total degradation occurred in one step for rPP composites due to radical chain mechanics and rapid chain scission [31] as compared to the four peaks for rHDPE composites. The complete decomposition of the polymer matrix of both polyolefins can be seen in the peaks within the range of 454 °C to 540 °C for rHDPE composites and 430 °C to 506 °C for rPP composites. The rice hull composites made with <0.5 mm fibers degraded at slightly higher temperatures, showing that they were more thermally stable. The small weight loss at the beginning of the rPP plots could possibly represent the breakdown of rice-hulls components from 345 °C to 420 °C. Raghu et al. [25] observed the breakdown of virgin polypropylene between 400 °C and 480 °C and increased degradation in the composite at about 500 °C. The weakening of the cellulosic fibers of the rHDPE can be seen in the peak range between 317 °C to 423 °C with the possible presence of volatile impurities at the small peak at 303 °C and 299 °C for <0.5 mm and <1 mm, respectively. 

### 3.4. FTIR Analysis

**Figure 4:**
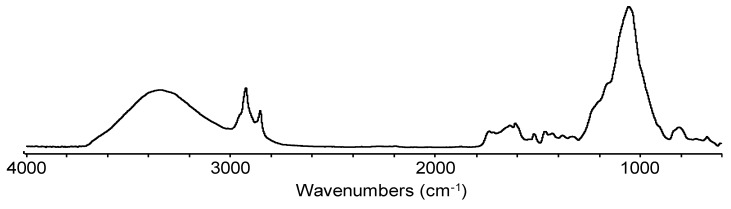
Fourier-transform infrared spectroscopy (FTIR) spectrum of rice hulls.

**Figure 5:**
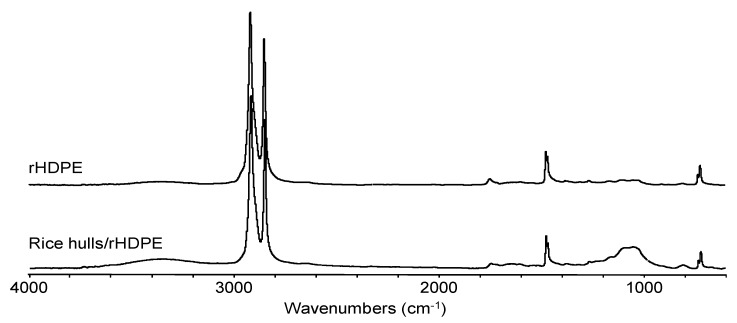
FTIR of recycled HDPE (top) and rice hulls (<0.5 mm)/rHDPE composite (bottom).

The FTIR spectra of the rice husk fiber are shown in Figure 4. The spectrum shows the presence of the hydroxyl group (-OH) in cellulosic fibers with a stretched medium intensity broad band at 3334 cm^−1^, C–H stretching bands at 2921 cm^−1^ and 2851 cm^−1^ associated with lignin and lipids, and two bands at 1603 cm^−1^ and 1513 cm^−1^ assigned to aromatic skeletal vibrations of lignin [11]. A high intensity band at 1048 cm^−1^ is assigned to the C–O stretching of cellulose and hemicellulose [32]. The rice hull (<0.5mm) /rHDPE composite spectra (Figure 5) shows bands at a lower intensity than the pure rice hulls due to dilution.

**Figure 6:**
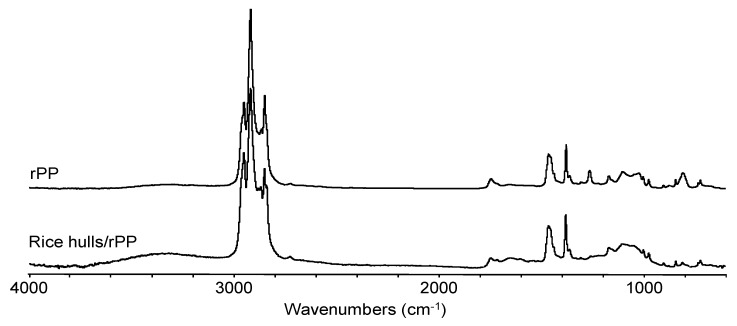
FTIR spectra of recycled PP (top) and rice hulls (<0.5 mm)/rPP composite (bottom).

Major bands for rPP (Figure 6) were between 2866 and 2949 cm^−1^ assigned to C–H stretching vibrations. In addition, bands for –CH_2_ and –CH_3_ groups were observed at 1454 cm^−1^ and 1375 cm^−1^ respectively and are characteristic for the propylene unit. The band at 803 cm^−1^ was assigned to a C=C bond that could be due to some thermal degradation [33]. This band could help explain the presence of the minor peak found in the DSC analysis for rPP. After extrusion and compounding of the rice hulls with rPP, the C=C band was shown to decrease (Figure 6). 

### 3.5. Melt Flow and Rheological Properties

Due to the reprocessing of the polyolefins, a minimal temperature of 190 °C was used to reduce the thermal degradation bracket and chain breaking as studied by Oblak et al. [19]. The melt flow rate (MFR), which is an industrial test, was determined and compared to rheology results (Table 4). Under the applied temperature, load conditions of 2.15 kg and 15 kg for polyolefins and composites were determined, respectively. The MFR for rHDPE composites were 4.6 and 3.9 g/10 min with corresponding viscosities of 15.9 and 18.6 kPa.s for <1 mm and <0.5 mm particles, respectively. The MFR of rHDPE <0.5 mm particle size is comparable with the MFR of corn cob composites as reported by Adefisan and McDonald [34]. In the presence of natural fiber fillers, there is the resistance to flow and sliding between the intermolecular layers, causing an increase in melt viscosity [17]. rPP composites MFR were significantly higher, with values of 125 and 109 g/10 min and concomitant lower viscosities of 0.35 and 0.41 kPa.s for <1 mm and <0.5 mm particles, respectively. rPP experienced a high displacement during the MFR process due to the reduction in entanglements and molar mass [19]. 

**Figure 7:**
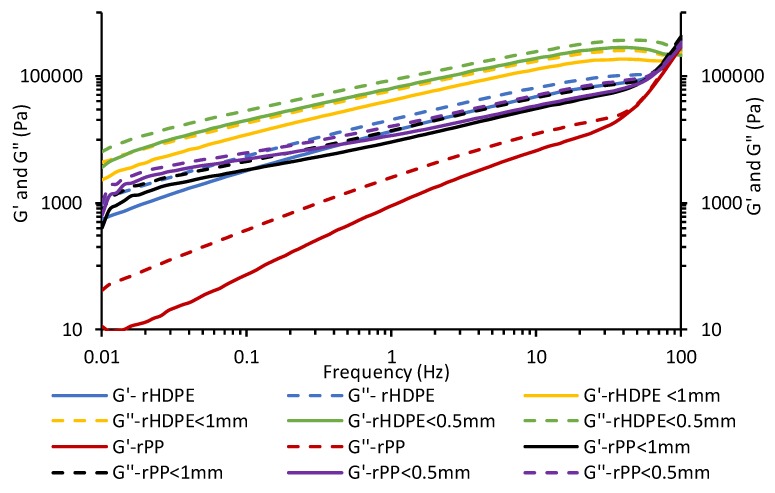
Dynamic rheological measurements (G’ and G”) of polyolefins and rice hull composites.

Dynamic rheology tests were performed to study the influence of shear rate on the melt (Figure 7). An increase in viscous modulus (G’’) for polyolefins and composites with frequency was observed, indicating good viscoelastic liquid behaviors of the polyolefins in the blend and strong-structured matrices. The probability of energy storage in the composites is observed via the elastic modulus (G’) [18]. The addition of the rice hulls also caused an increase in the G’ and G’’, and reduced the spacing for rPP at lower frequency. Differences in G’ are also observed with each composite due to the varying particle sizes and polymer matrices. rPP composites exhibited the lowest G’–G’’ curves in comparison to the rHDPE composites. 

**Figure 8:**
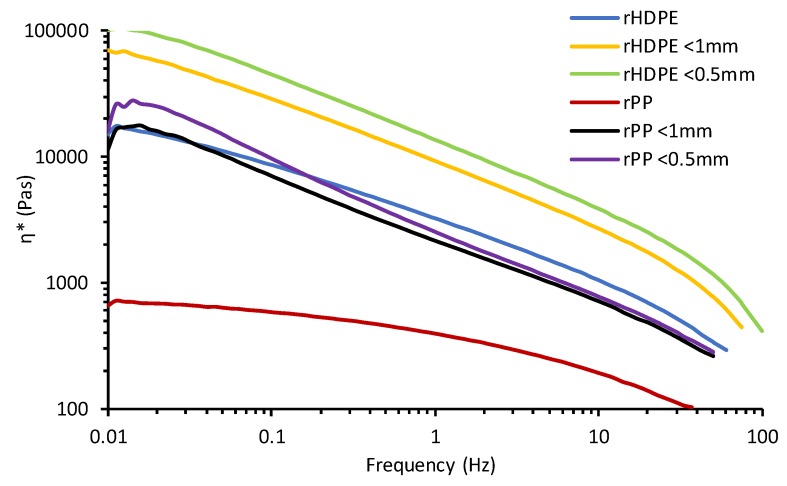
Flow curves of complex viscosity (η*) of polyolefins and rice hull composites with frequency.

Complex viscosity (η*) also decreased (Figure 8) from low to high frequency for all samples as a result of shear thinning [35]. rPP and its composites had a lower η* than rHDPE, which had a better cross-linked structure in composite melts [36]. With multiple extrusion and processing cycles for PP, a rapid decrease in η* was experienced [20]. The highest η* of the <0.5 mm for each case of composite was due to the high surface area and better interactivity in the composites. This increase in η* for the <0.5mm composites also corresponded with the results from the MFR. With the heat applied to the materials, the higher η* of the composites occurred because of lower heat transfer in the insulating-like rice hulls as compared to the polyolefins [13]. Melt flow rate, apparent and complex viscosity also conform statistically to measured results showing a significant difference with the addition of different sized fibers as seen in Table 4. 

**Figure 9:**
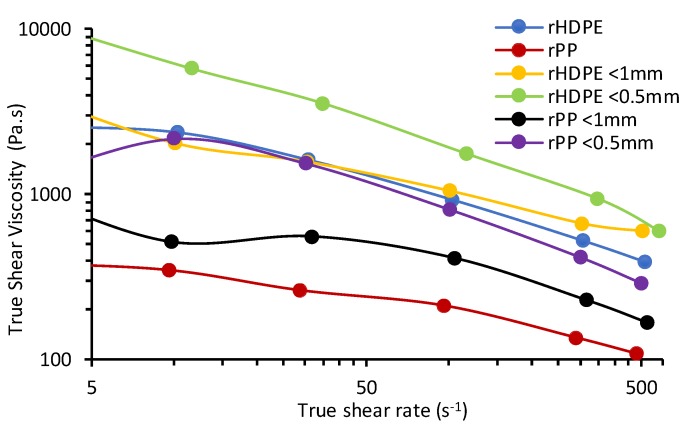
Flow curves of true shear viscosity versus true shear rate by capillary rheology of polyolefins and rice hull composites.

In contrast to dynamic rheology, which runs at lower frequencies, capillary rheology was used to study the flow behavior and the response of polymer matrices to higher shear rates [37]. Shear rates applied were in the range of 2.9 s^−1^ to 580 s^−1^ for both polyolefins and composites. A gradual decrease in shear viscosity was observed with increased shear rates due to shear thinning behavior which also seen in the dynamic rheology results. Data was Bagley and Rabinowitch corrected, according to ASTMD3835-02, for both dies used to reduce the effects of shear thinning and to improve viscosity results. This yielded corresponding results of true viscosity for both dies. True shear viscosity vs. shear rate plots are shown in Figure 9 for polyolefins and composites. Shear viscosity values were within 10^2^–10^4^ Pa.s for rHDPE and composites and 10^2^–10^3^ Pa.s for rPP composites. A lower range of results (10^1^–10^3^ Pa.s) were attained by Mazzanti et al. [38]. Rice hulls (<0.5 mm)/rHDPE composite registered the highest viscosity when compared to other composites and polyolefins due to density effects, surface area and thermal stability. At low crosshead speeds, rPP composites experienced breaking, which accounted for the non-linear results attained. A brittle effect from cooled extrudate of rPP composites was observed, which could have been caused by the continuous degradation from multiple extrusion cycles [39]. The shear viscosity from capillary rheology in both cases were significantly higher than that from dynamic rheology when both were compared. A similar trend is also seen in the study done by Mazzanti et al. [38]. Extrudate swells were also measured, and the 27 mm die extrudates were smaller in diameter for all composites and polyolefins in comparison to the 14 mm die. There was a minimal increase in swell ratio of the polypropylene composites at increased crosshead speed. 

### 3.6. Water Absorption

**Figure 10:**
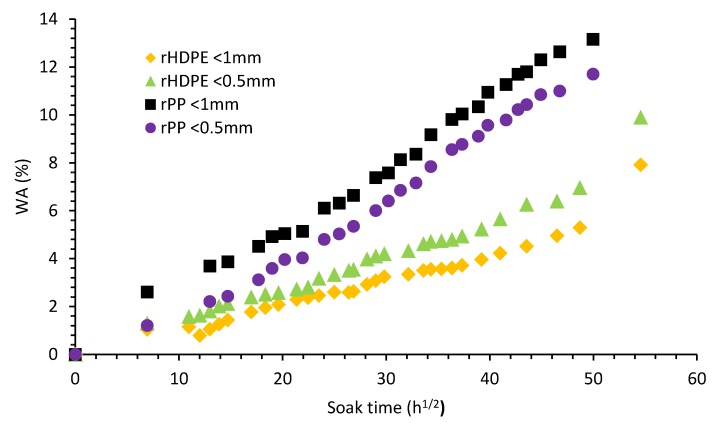
Water soak of polyolefin composites as a function of time.

The relationship of water uptake with time for the composites was analyzed based on Fick’s diffusion behavior [40] and, an initial increase in water absorption was observed for both composites as seen in Figure 10 with corresponding diffusivities. The respective WA for rice hulls <1 mm and <0.5 mm composites were 9.9% and 7.8% for rHDPE and 13.2% and 11.7% for rPP (Table 5). The presence of the large <1mm particle size created a larger exposed hydrophilic surface with the potential for more voids which would absorb more moisture over time [41]. The D_f_ for the <1 mm and <0.5 mm composites were 3.2 × 10^−11^ m^2^/s and 3.5 × 10^−11^ m^2^/s for rHDPE and 6.1 × 10^−11^ m^2^/s and 7.0 × 10^−11^ m^2^/s for rPP, respectively. Since a higher diffusivity signifies a shorter time to approach equilibrium absorption [42], <0.5 mm composites for polypropylene will absorb less water over more time. The relationship between water absorption and fiber size for both composites were significantly different. 

## 4. Conclusions

Rice-hulls natural fiber of <1 mm and <0.5 mm particle sizes were successfully compounded with recycled polyolefins, rHDPE and rPP, using a twin-screw extruder to form extruded profiles. Rheology, mechanical, and thermal characterization were used to analyze the composites, and were shown to have a performance comparable to WPC, and hence could be a direct substitute. Rheology was performed via dynamic and capillary methods with a minimum temperature of 190 °C to reduce the adverse effect of thermal degradation. Although the densities of the smaller particle-sized composites were low, stability results were not negatively affected. The <0.5 mm composites of rHDPE possessed better thermal stability and tensile properties when compared to rPP composites and raw recycled polyolefins, due to the size and incorporation of rice-hull fibers. This study affirms the importance of upcycling plastic waste and natural fiber residues into sustainable composite products for industrial and residential applications. 

## Figures and Tables

**Table 1:** Density and tensile properties of the polyolefins and rice hull composites.

Polyolefin	Composite Particles	Density kg/m^3^	Strength (MPa)	Modulus (GPa)	EAB (J)
**rHDPE**	-	978 (0.002) ^c^	26.0 (0.5) ^b^	1.1 (0.1) ^b^	3.64 (0.4) ^c^
	<1 mm	1157.4 (3.1) ^a^	25.1 (2.9) ^b^	2.7 (0.1) ^a^	0.65 (0.22) ^a^
	<0.5 mm	1109.9 (1.1) ^b^	26.3 (2.0) ^a^	3.1 (0.5) ^a^	0.64 (0.16) ^b^
**rPP**	-	912 (0.001) ^b^	14.3 (1.9) ^b^	1.7 (0.2) ^b^	0.16 (0.06) ^c^
	<1 mm	1122.6 (5.9) ^c^	14.5 (0.3) ^a^	2.9 (0.5) ^a^	0.12 (0.02) ^b^
	<0.5 mm	1109.3 (1.7) ^a^	16.7 (0.5) ^a^	3.5 (0.2) ^a^	0.19 (0.05) ^a^

Note: Standard deviations are in parentheses. Each polyolefins’ sample with different superscript letters (^a, b, c^) are statistically different (*p* < 0.05) via a one-tailed *t*-test.

**Table 2:** Thermogram Peak data for polyolefins and corresponding rice hull composites.

		DSC				TMA
Polyolefin	Composite Particles	T_m_ (°C)	T_c_ (°C)	X_c_ (%)	ΔH_c_ (J/g)	T_m_ or T_s_ (°C)
**rHDPE**	-	135.1 (0.14)	118.9 (0.16)	143.7 (2.09)	203.2 (2.65)	130.1
	<1 mm	134.0 (0.09)	119.3 (0.04)	71.6 (0.89)	99.2 (2.76)	130.2
	<0.5 mm	133.0 (0.73)	119.6 (0.21)	61.9 (0.82)	85.4 (1.42)	131.3
**rPP**	-	125.1 (0.08) ^a^	112.3 (0.04) ^a^	-	-	-
		163.8 (0.06) ^b^	123.8 (0.06) ^b^	65.5 (0.26)	67.0 (0.41)	156.4
	<1 mm	125.3 (0.05) ^a^	113.6 (0.02) ^a^	-	-	-
		162.9 (0.04) ^b^	123.2 (0.05) ^b^	33.3 (0.29)	33.7 (0.39)	157.7
	<0.5 mm	125.4 (0.11) ^a^	113.9 (0.03) ^a^	-	-	-
		163.2 (0.09) ^b^	123.4 (0.09) ^b^	32.2 (0.27)	32.1 (0.72)	161.2

Note: Standard deviations are in parentheses. ^a^ minor peaks and ^b^ major peaks.

**Table 3:** Thermal degradation behavior of polyolefin composites.

Polyolefin	Composite Particles	Onset (°C)	1st Peak (°C)	2nd Peak (°C)	3rd Peak (°C)	Final Decomposition (°C)
**rHDPE**	<1 mm	278	299	380	502	512
	<0.5 mm	276	303	383	493	525
**rPP**	<1 mm	322	-	-	-	472
	<0.5 mm	320	-	-	-	493

**Table 4:** Melt flow and Rheology results for polyolefins and rice hull composites.

				Dynamic	Capillary	
Polyolefin	Composite Particles	MFR (g/10 min)	Apparent Viscosity (kPa.s)	η* at 1.06 Hz (kPa.s)	Apparent Viscosity at 30 s^−1^ (Pa.s)	True Viscosity at 30 s^−1^ (Pa.s)
**rHDPE**	-	0.961 (0.023) ^c^	10.91 (0.260) ^c^	3.17 (1.32) ^c^	2387	1621
	<1 mm	4.592 (0.021) ^b^	15.91 (0.073) ^a^	8.95 (0.816) ^b^	7091	1580
	<0.5 mm	3.919 (0.010) ^a^	18.65 (0.039) ^b^	13.34 (1.56) ^a^	6148	3548
**rPP**	-	25.0 (2.318) ^c^	0.38 (0.032) ^b^	0.39 (0.014) ^c^	376	263
	<1 mm	124.9 (1.278) ^a^	0.35 (0.004) ^b^	2.09 (0.300) ^a^	850	557
	<0.5 mm	109.4 (0.062) ^b^	0.41 (0.006) ^a^	2.48 (0.240) ^b^	808	1544

Note: Standard deviation in parentheses. Each polyolefins’ sample with different letters (a, b, c) are statistically different (*p* < 0.05) via a one-tailed *t*-test

**Table 5:** Water soak properties of polyolefin composites.

		WA (%)		Diffusion Coefficient
Polyolefin	Composite Particles	2 Days	100 Days	m^2^/s (10^−11^)
**rHDPE**	<1 mm	1.3 (0.3) ^a^	9.9 (5.5) ^b^	3.2
	<0.5 mm	1.1 (0.3) ^a^	7.8 (0.9) ^a^	3.5
**rPP**	<1 mm	2.6 (1.1) ^a^	13.2 (0.6) ^a^	6.1
	<0.5 mm	1.2 (1.1) ^a^	11.7 (1.3) ^b^	7.0

Note: Standard deviation in parentheses. Each polyolefins’ sample with different superscript letters (^a, b, c^) are statistically different (*p* < 0.05) via a one-tailed *t*-test.

## References

[1] Oza S., Wang R., Lu N. (2011). Thermal and mechanical properties of recycled high-density polyethylene/hemp fiber composites. Int. J. Appl. Sci. Technol..

[2] Hong H., Li X., Liu H., Zhang H., He H., Xu H., Jia D. (2016). Transform rice husk and recycled polyethylene into high performance composites: Using a novel compatibilizer to infiltratively enhance the interfacial interactions. Prog. Rubber Plast. Recycl. Technol..

[3] Atikler U., Basalp D., Tihminlioğlu F. (2006). Mechanical and morphological properties of recycled high-density polyethylene filled with calcium carbonate and fly ash. J. Appl. Polym. Sci..

[4] Chirayil C.J., Joy J., Maria H.J., Krupa I., Thomas S. (2016). Polyolefins in Automotive Industry. Polyolefin Compounds and Materials.

[5] Parmar H.B., Gupta R.K., Bhattacharya S.N. (2009). Rheological and molecular properties of organic peroxide induced long chain branching of recycled and virgin high density polyethylene resin. Polym. Eng. Sci..

[6] Zahavich A.T.P., Latto B., Takacs E., Vlachopoulos J. (1997). The effect of multiple extrusion passes during recycling of high density polyethylene. Adv. Polym. Technol. J. Polym. Process. Inst..

[7] Abad M.J., Ares A., Barral L., Cano J., Diez F.J., García-Garabal S., Ramirez C. (2004). Effects of a mixture of stabilizers on the structure and mechanical properties of polyethylene during reprocessing. J. Appl. Polym. Sci..

[8] Enpower Corp. https://www.enpowercorp.com/facilities/wadham-facility/.

[9] Rahman M.R., Islam M.N., Huque M.M., Hamdan S., Ahmed A.S. (2010). Effect of chemical treatment on rice husk (RH) reinforced polyethylene (PE) composites. BioResources.

[10] Arjmandi R., Hassan A., Majeed K., Zakaria Z. (2015). Rice husk filled polymer composites. Int. J. Polym. Sci..

[11] Chen R.S., Ab Ghani M.H., Ahmad S., Salleh M.N., Tarawneh M.A.A. (2015). Rice husk flour biocomposites based on recycled high-density polyethylene/polyethylene terephthalate blend: Effect of high filler loading on physical, mechanical and thermal properties. J. Compos. Mater..

[12] Freire C.S.R., Silvestre A.J.D., Neto C.P., Belgacem M.N., Gandini A. (2006). Controlled heterogeneous modification of cellulose fibers with fatty acids: Effect of reaction conditions on the extent of esterification and fiber properties. J. Appl. Polym. Sci..

[13] Gao H., Xie Y., Ou R., Wang Q. (2012). Grafting effects of polypropylene/polyethylene blends with maleic anhydride on the properties of the resulting wood–plastic composites. Compos. Part A: Appl. Sci. Manuf..

[14] Zhou X., Yu Y., Lin Q., Chen L. (2013). Effects of maleic anhydride-grafted polypropylene (MAPP) on the physico-mechanical properties and rheological behavior of bamboo powder-polypropylene foamed composites. BioResources.

[15] Hornsby P.R. (1999). Rheology, Compounding and Processing of Filled Thermoplastics. Mineral Fillers in Thermoplastics I.

[16] Habibi M., Najafi S.K., Ghasemi I. (2017). Rheological and mechanical properties of composites made from wood flour and recycled LDPE/HDPE blend. Iran. Polym. J..

[17] Sewda K., Maiti S.N. (2012). Effect of bark flour on melt rheological behavior of high density polyethylene. J. Appl. Polym. Sci..

[18] Ogah A.O., Afiukwa J.N., Nduji A.A. (2014). Characterization and comparison of rheological properties of agro fiber filled high-density polyethylene bio-composites. Open J. Polym. Chem..

[19] Oblak P., Gonzalez-Gutierrez J., Zupančič B., Aulova A., Emri I. (2015). Processability and mechanical properties of extensively recycled high density polyethylene. Polym. Degrad. Stab..

[20] Da Costa H.M., Ramos V.D., Rocha M.C. (2005). Rheological properties of polypropylene during multiple extrusion. Polym. Test..

[21] Jin H., Gonzalez-Gutierrez J., Oblak P., Zupančič B., Emri I. (2012). The effect of extensive mechanical recycling on the properties of low density polyethylene. Polym. Degrad. Stab..

[22] Kuram E., Ozcelik B., Yilmaz F. (2016). The effects of recycling process on thermal, chemical, rheological, and mechanical properties of PC/ABS binary and PA6/PC/ABS ternary blends. J. Elastomers Plast..

[23] Adefisan O.O., McDonald A.G. (2017). Evaluation of Wood Plastic Composites Produced from Mahogany and Teak. Int. J. Adv. Eng. Res. Sci..

[24] Zykova A.A., Pantyukhov P.A., Kolesnikova N., Monakhova T., Popov A.A. (2018). Influence of filler particle size on physical properties and biodegradation of biocomposites based on low-density polyethylene and lignocellulosic fillers. J. Polym. Environ..

[25] Raghu N., Kale A., Chauhan S., Aggarwal P. (2018). Rice husk reinforced polypropylene composites: Mechanical, morphological and thermal properties. J. Indian Acad. Wood Sci..

[26] Rahman W.A.W.A., Isa N.M., Rahmat A.R., Adenan N., Ali R.R. (2010). Rice Husk/High Density Polyethylene Bio-Composite: Effect of Rice Husk Filler Size and Composition on Injection Molding Processability with Respect to Impact Property. Adv. Mater. Res..

[27] Wang X., Yu Z., McDonald A.G. (2019). Effect of Different Reinforcing Fillers on Properties, Interfacial Compatibility and Weatherability of Wood-plastic Composites. J. Bionic Eng..

[28] Yang H.S., Kim H.J., Son J., Park H.J., Lee B.J., Hwang T.S. (2004). Rice-husk flour filled polypropylene composites; mechanical and morphological study. Compos. Struct..

[29] Cui Y.H., Tao J., Noruziaan B., Cheung M., Lee S. (2010). DSC Analysis and Mechanical Properties of Wood—Plastic Composites. J. Reinf. Plast. Compos..

[30] Ndiaye D., Diop B., Thiandoume C., Fall P.A., Farota A.K., Tidjani A. (2012). Morphology and thermo mechanical properties of wood/polypropylene composites. Polypropylene.

[31] Kim H.-S., Yang H.-S., Kim H.-J., Park H.-J. (2004). Thermogravimetric analysis of rice husk flour filled thermoplastic polymer composites. J. Therm. Anal. Calorim..

[32] Chen D., Zhou J., Zhang Q. (2014). Effects of Torrefaction on the Pyrolysis Behavior and Bio-Oil Properties of Rice Husk by Using TG-FTIR and Py-GC/MS. Energy Fuels.

[33] Lin-Vien D., Colthup N.B., Fateley W.G., Grasselli J.G. (1991). The Handbook of Infrared and Raman Characteristic Frequencies of Organic Molecules.

[34] Adefisan O.O., McDonald A.G. (2001). Evaluation of the Flexural Strength, Sorption, Rheological and Thermal Properties of Corncob Plastic Composites. Int. J. Adv. Eng. Res. Sci..

[35] Yokozeki T., Carolin Schulz S., Buschhorn S.T., Schulte K. (2012). Investigation of shear thinning behavior and microstructures of MWCNT/epoxy and CNF/epoxy suspensions under steady shear conditions. Eur. Polym. J..

[36] Yin S., Tuladhar R., Shi F., Shanks R.A., Combe M., Collister T. (2015). Mechanical reprocessing of polyolefin waste: A review. Polym. Eng. Sci..

[37] Kajaks J., Kalnins K., Matvejs J. (2018). Rheological Properties of Wood-Plastic Composites Based on Polypropylene and Birch Wood Plywood Production Residues. Key Engineering Materials.

[38] Mazzanti V., Mollica F., El Kissi N. (2016). Rheological and mechanical characterization of polypropylene-based wood plastic composites. Polym. Compos..

[39] Premalal H.G., Ismail H., Baharin A. (2002). Comparison of the mechanical properties of rice husk powder filled polypropylene composites with talc filled polypropylene composites. Polym. Test..

[40] Adefisan O.O., McDonald A.G. (2019). Evaluation of the strength, sorption and thermal properties of bamboo plastic composites. Maderas. Cienc. Y Tecnol..

[41] Bouafif H., Koubaa A., Perré P., Cloutier A., Riedl B. (2009). Wood particle/high-density polyethylene composites: Thermal sensitivity and nucleating ability of wood particles. J. Appl. Polym. Sci..

[42] Luo S., Cao J., McDonald A.G. (2017). Esterification of industrial lignin and its effect on the resulting poly (3-hydroxybutyrate-co-3-hydroxyvalerate) or polypropylene blends. Ind. Crops Prod..

